# The Analysis of the Glycosyltransferase Activity Gene Family in *Gossypium hirsutum* and Functional Verification of *GTs* Conferring Resistance to Verticillium Wilt

**DOI:** 10.3390/ijms26073170

**Published:** 2025-03-29

**Authors:** Mingli Zhang, Fang Zhu, Guo Sun, Yingjie Mi, Xuekun Zhang, Sifeng Zhao, Yu Yu, Hui Xi

**Affiliations:** 1 Key Laboratory of Oasis Agricultural Pest Management and Plant Protection Resources Utilization, College of Agriculture, Xinjiang Uygur Autonomous Region, Shihezi University, Shihezi 832003, China; zhangmingli4128@163.com (M.Z.); 15630077211@163.com (Y.M.); zhangxk2459@163.com (X.Z.); zhsf_agr@shzu.edu.cn (S.Z.); 2 Xinjiang Academy of Agricultural Reclamation Sciences, Shihezi 832003, China

**Keywords:** Glycosyltransferases, Verticillium wilt, *Gossypium hirsutum*, *GhGT61*

## Abstract

Glycosyltransferases (*GTs*) play an important role in plant growth and development, as well as responses to biotic and abiotic stresses. However, the function of the *GT* family in cotton resistance to Verticillium wilt is limited. In the present study, transcriptome analysis revealed eight *GTs* upregulated in susceptible cotton varieties and downregulated in resistant cotton varieties during early *Verticillium dahliae* inoculation, indicating they were involved in regulating the infection of *V. dahliae* in cotton. Promoter analysis revealed a high prevalence of MeJA (methyl jasmonate) and ABA (abscisic acid)-related cis-acting elements among these *GTs*. Genome-wide and location analysis of the homologous genes showed that these *GTs* were relatively conserved in evolution. Furthermore, a Virus-Induced Gene Silencing (VIGS) experimental results demonstrated a reduction in disease resistance after *GhGT61* silencing. These insights not only deepen our understanding of the *GT* family’s role in cotton, but also provide a foundation for future research on the disease resistance mechanisms of these genes.

## 1. Introduction

Cotton is a pivotal economic crop and the primary source of natural fiber for the textile industry [1]. However, it is severely affected by Verticillium wilt caused by *V. dahliae*. In China, this disease affects over 50% of cotton fields annually, resulting in substantial economic losses [2]. *V. dahliae* is particularly difficult to control due to its persistence in soil as dormant microsclerotia [3]. Currently, there is no effective chemical control for cotton Verticillium wilt [4]. The most cost-effective and efficient strategy involves the breeding and deployment of disease-resistant cotton varieties [5]. The scarcity of available resistance genes for genetic engineering has significantly constrained cotton molecular breeding.

Some natural glycosides or sugar esters with a more stable structure are usually formed by glycosylation modification [6]. Glycosylation is crucial for promoting plant storage, intracellular transport, and regulating the balance of growth agents within plants [7]. *GTs* can link sugar molecules to specific receptors and produce glycosidic bonds, which are present in most organisms and can be particularly important in plants [8]. One important role of *GTs* is as catalyzed plant glycosylation [9]. *GTs* transfer activated sugars to various plant molecules, including monosaccharides, oligosaccharides, polysaccharides, and non-carbohydrate substrates such as proteins, antibiotics, and lipids, leading to the glycosylation of these compounds [10]. *GTs* are essential for plant growth, development, and responses to biotic and abiotic stresses.

*GTs* play crucial roles in plant defense responses by regulating resistance pathways and glycosylation. For instance, AtUGT73C7 is a glycosyltransferase that regulates *Arabidopsis thaliana* resistance to *Pseudomonas syringae* by redirecting the phenylpropanoid pathway and activating the Toll-interleukin-1 receptor (TIR)-type nucleotide-binding leucine-rich repeat (NLR) protein SNC1 [11]. *AtUGT76B1* could regulate the basic defense and systemic acquired resistance (SAR) by coordinating the glycosylation of three defense activators, such as salicylic acid (SA), isoleucic acid, and N-hydroxypipecolic acid [12]. Additionally, *TaUGT6* is a novel UDP-glycosyltransferase gene that enhances wheat resistance to Fusarium head blight by converting deoxynivalenol (DON) into DON-3-glucoside [13]. A recent study also showed that *CsUGT87E7* encodes a SA carboxyltransferase that enhances the disease resistance of tea plants to *Pseudopestalotiopsis camelliae-sinensis* by regulating SA homeostasis [14]. These studies demonstrate that *GTs* have significant roles in disease resistance, whereas there have been few reports on the role of *GTs* in cotton against *V. dahliae*.

In our study, we found that *GTs* are involved in the regulation of cotton’s defense response to *V. dahliae*. In order to clarify the molecular function of *GTs* in cotton, the *GTs* were analyzed in *G. arboreum*, *G. raimondii*, *G. hirsutum*, and *G. barbadense* genomes, respectively. The characteristics of *GTs* were also comprehensively analyzed in *G. hirsutum*, including their chromosomal locations, motif distributions, gene structures, and the cis-elements in the promoter regions. Additionally, the impact of *GhGT61* on *V. dahliae* was verified by VIGS. This study will preliminarily clarify the function of *GTs* and provide gene resources for molecular breeding of upland cotton resistant to Verticillium wilt.

## 2. Results

### 2.1. GTs Are Involved in the Response of Cotton to V. dahliae Infection

**Figure 1:**
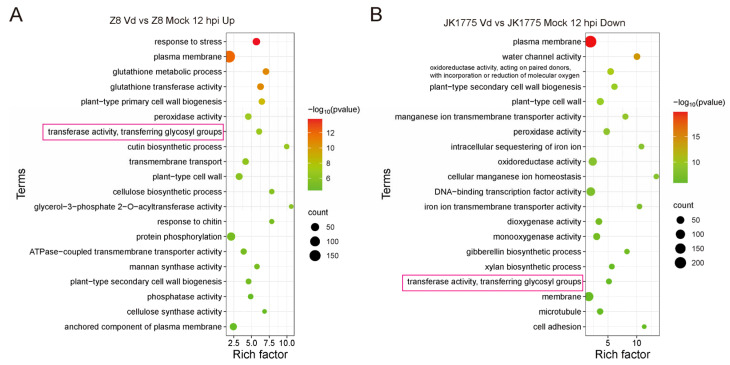
GO analysis of DEGs in response to *V. dahliae* infection at 12 hpi in resistant and susceptible upland cotton. (**A**) GO analysis of up-regulating DEGs in response to *V. dahliae* infection at 12 hpi in susceptible upland cotton Z8. (**B**) GO analysis of down-regulating DEGs in response to *V. dahliae* infection at 12 hpi in resistant upland cotton JK1775. The transferase activity and transferring glycosyl group pathway were marked with red boxes.

In order to screen the key resistance-associated genes of upland cotton responding to *V. dahliae* infection, we analyzed the differentially expressed genes (DEGs) in both resistant and susceptible upland cotton varieties after inoculation with *V. dahliae*. Gene Ontology (GO) enrichment analysis revealed that the transferase activity and transferring glycosyl group pathway were significantly enriched in both cotton varieties at 12 h post-inoculation. However, the expression patterns of these DEGs were completely opposite because the related DEGs were up-regulated and down-regulated in susceptible and resistant upland cotton, respectively (Figure 1A,B).

**Figure 2:**
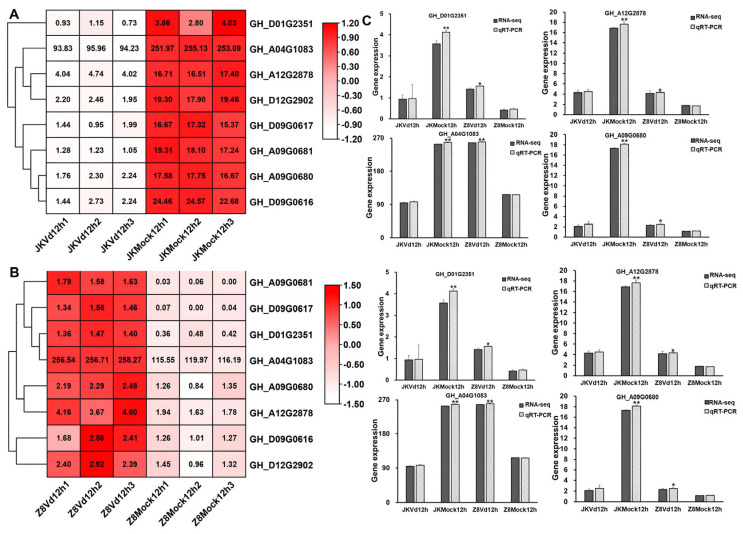
Expression analysis of eight *GTs* in response to *V. dahliae* infection in resistant and susceptible upland cotton at 12 hpi. (**A**) Heatmap of eight *GTs* in resistant upland cotton JK1775 in response to *V. dahliae* infection at 12 hpi. (**B**) Heatmap of eight *GTs* in susceptible upland cotton Z8 in response to *V. dahliae* infection at 12 hpi. (**C**) qRT-PCR verification of *GTs* transcriptome data. The data are mean ± SD (*n* = 3). Significance is determined using Duncan’s multiple range test, indicated by * *p* ≤ 0.05 and ** *p* ≤ 0.01.

There were eight identical *GTs* involved in the transferase activity and transferring glycosyl group pathway in both the resistant and susceptible upland cotton (Figure 2A,B). These *GTs* may play a crucial role in the immune response of upland cotton to *V. dahlia.* In order to verify the reliability of the selected *GT* data, the differential expression levels of *GTs* were analyzed by qRT-PCR in the early stage of *V. dahliae* infection in both resistant and susceptible upland cotton. The results indicated that the expression level of the *GTs* were basically consistent with the results of the transcriptome data (Figure 2C). Notably, *GH_A04G1083* exhibited a high expression in both the inoculated and uninoculated upland cotton, with expression levels higher than those of other *GTs* (Figure 2). These results suggested that *GH_A04G1083* may play a role not only in the immune response to *V. dahliae,* but also in other vital activities of upland cotton.

### 2.2. Domain Structure and Genetic Relationship of the Eight GTs in Cotton

**Figure 3:**
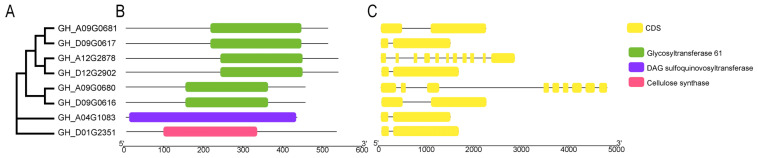
Phylogenetic tree, gene structure, and conservative motif analysis of eight *GTs*. (**A**) Neighbor-joining phylogenetic tree of eight *GTs*. (**B**) Prediction of conserved motifs in the *GTs*, with different colors representing distinct motifs. (**C**) Analysis of the intron-exon structure of the *GTs*, where exons and introns are depicted by yellow boxes and gray lines, respectively.

To elucidate the biological function of the eight *GTs* further, we analyzed their domain structure and genetic relationship. The results revealed that although the distribution of CDS sequences on the eight *GTs* showed certain differences (Figure 3C), *GH_A09G0681* and *GH_D09G0617*, *GH_A12G2878* and *GH_D12G2902*, and *GH_A09G0680* and *GH_D09G0616* were clustered together, respectively, indicating a relatively close relationship (Figure 3A). These six *GTs* were identified as members of glycosyltransferase family 61 based on the domain analysis (Figure 3B). Additionally, the *GH_D01G2351* and *GH_AO4G1083* were classified under glycosyltransferase family 1 and glycosyltransferase family 2, respectively (Figure 3A).

**Figure 4:**
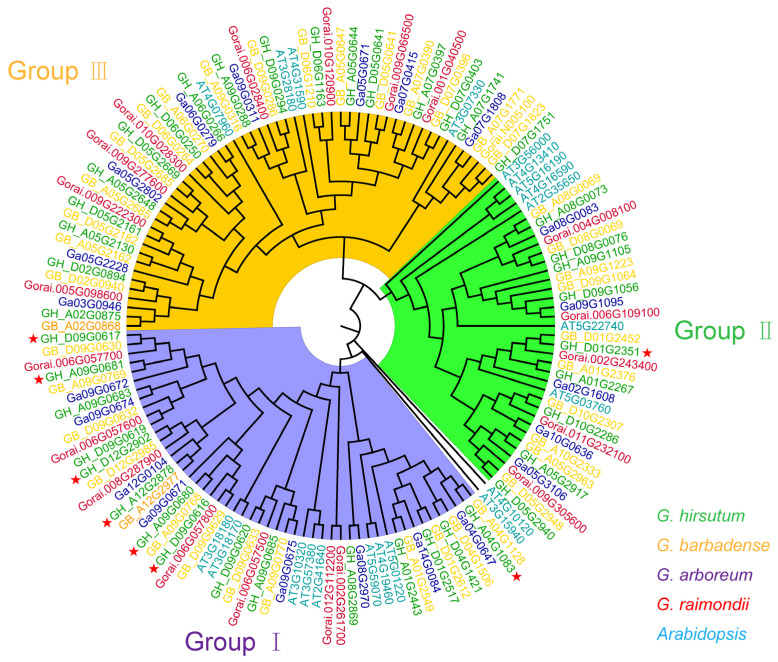
Phylogenetic analysis of *GTs* proteins from *G. hirsutum*, *G. barbadense*, *G. arboreum*, *G. raimondii*, and *A. thaliana*. The *GTs* proteins from the five species are indicated by different colors. The phylogenetic tree is constructed using MEGA X with the maximum likelihood method and 1000 bootstrap replications. Eight *GTs* were marked with red stars.

To investigate the evolutionary relationship of the eight *GTs* in cotton, we obtained their homologous genes in *G. arboreum*, *G. raimondii*, *G. hirsutum*, *G. barbadense,* and *A. thaliana* using the Basic Local Alignment Search Tool (BLAST, version 2.16) in cotton and *A. thaliana* genomes. The phylogenetic analysis revealed that their homologous *GTs* were divided into three distinct groups. Specifically, seven *GTs* were classified into Group Ι, while one *GT* (GH_D01G2351) fell into Group II (Figure 4). Notably, the number of homologous *GTs* in *G. barbadense* and *G. hirsutum* was approximately twice than that of *G. arboreum* and *G. raimondii* (Figure 4). These results suggest that these *GTs* may have undergone different differentiation paths and selection pressures during evolution, resulting in the formation of gene families with diverse functions and characteristics.

### 2.3. Analysis of Promoter Region of the Eight GTs

**Figure 5:**
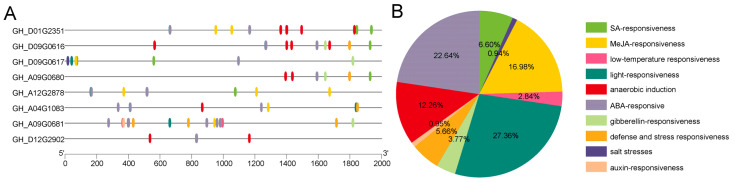
Cis-acting elements of *GTs* in upland cotton. (**A**) Predicted cis-elements in the promoter regions of *GTs*. (**B**) Proportion of different types of cis-elements. Ten groups of cis-acting elements are identified and represented by color-coded boxes.

Promoter regions are crucial for determining the gene expression patterns and functions. In order to predict the roles of *GTs*, we analyzed the upstream cis-acting elements within their promoter regions. Interestingly, many of these elements were associated with plant hormones, including ABA, gibberellin (GA), SA, and MeJA, as well as abiotic stress factors such as anaerobic conditions, defense and stress responses, light responses, and low-temperature responses (Figure 5A). Among these elements, hormone-related elements (SA, MeJA, auxin, ABA, and gibberellin) account for a significant proportion, representing 50.93% of the total elements (Figure 5B). More importantly, defense and stress response elements accounted for 5.66% of the total (Figure 5B). Notably, each *GT* contains at least one hormone-related cis-acting element, particularly those related to ABA. Among the eight *GTs*, *GH_A09G0680*, *GH_D09G0616*, and *GH_D12G2902* lack MeJA-related elements. Collectively, these results indicate that *GTs* are crucial for conferring resistance to upland cotton against adverse environmental conditions.

### 2.4. Chromosomal Localization of Cotton GTs

**Figure 6:**
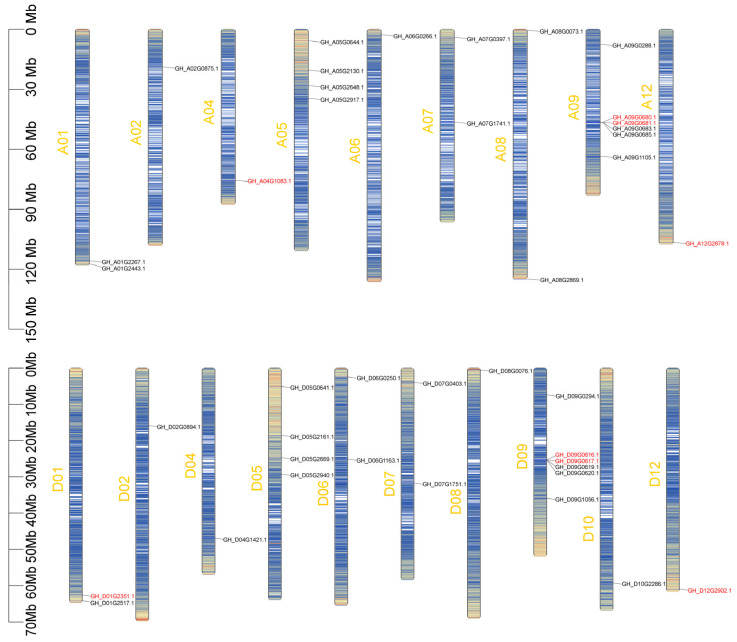
Chromosome distribution of *GTs* in *G. hirsutum*. The heatmap visualizes the chromosome density, with a blue-to-red color scale representing low to high-density values. The chromosome number is annotated along the left side in orange, while eight *GTs* (red) and their homologous genes (black) are labeled on the right flanking axis, respectively.

To investigate the genomic distribution of cotton *GTs*, a chromosome map was constructed for 41 *GTs* in upland cotton. A total of 20 *GTs* were mapped to 9 chromosomes of the At subgenome, while 21 *GTs* were mapped to 10 chromosomes of the Dt subgenome (Figure 6). The distribution of *GTs* across At and Dt chromosomes is uneven, ranging from 1 to 6. Specifically, there are 6 *GTs* located on chromosomes A09 and D09, while 6 *GTs* are located on chromosomes A02, A04, A06, A12, D02, D04, D06, D10, and D12. Notably, the number and chromosomal location of certain *GTs* on the At subgenome are the same as those on the Dt subgenome, as evidenced by pairs such as A01-D01, A02-D02, A04-D04, A05-D05, A07-D07, A09-D09, and A12-D12.

### 2.5. Synteny Analysis of GTs in Cotton

**Figure 7:**
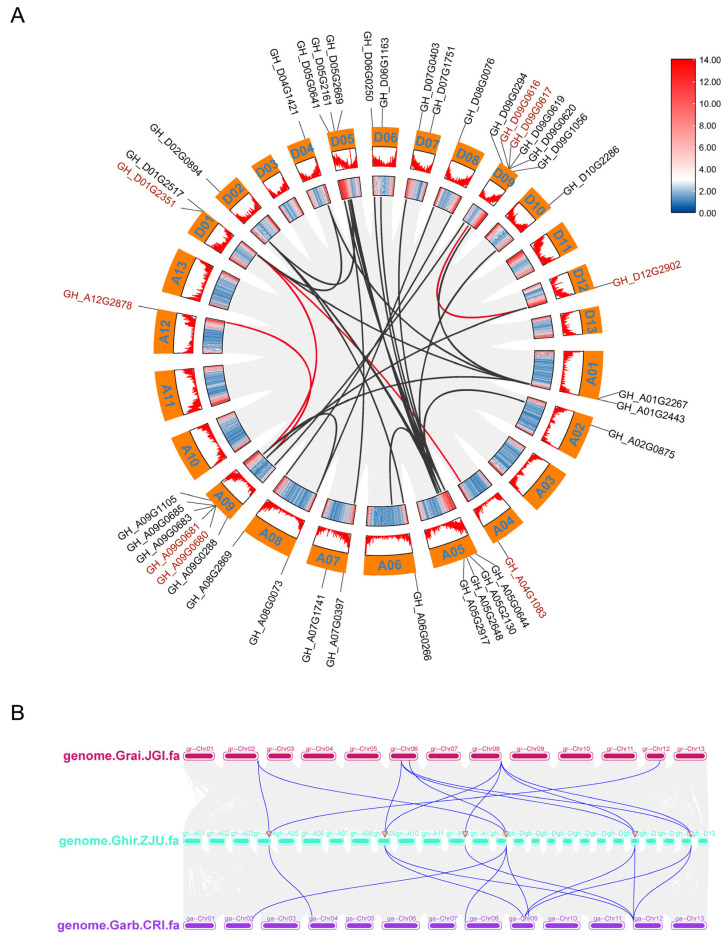
Intraspecific and interspecific collinearity analysis of eight *GTs*. (**A**) Synteny analysis between At and Dt subgenomes in *G. hirsutum*. Duplicated gene pairs are denoted by red connecting lines, whereas gray lines indicate identical gene pairs genome-wide. Eight *GTs* are labeled in red, with homologous genes distinguished by black labeling. (**B**) Synteny of repetitive gene pairs in three cotton species (*G. hirsutum*, *G. arboretum,* and *G. raimondii*).

In order to investigate the fragment replication and tandem replication of *GTs* in upland cotton, the amplification mechanism of cotton *GTs* were analyzed using BLAST, version 2.16 and MCScanX, https://github.com/wyp1125/MCScanX, accessed on 13 August 2024. The results suggested that six of the eight *GTs* formed gene clusters, including *GH_A04G1083*-*GH_D01G2517*, *GH_D01G2351*-*GH_A09G0685*, *GH_A12G2878*-*GH_A09G0680*, and *GH_D09G0616*-*GH_D12G2902*, which constituted four tandem clusters on chromosomes A12, A09, D09, and D12, respectively (Figure 7A). In contrast, *GH_D09G0617* and *GH_A09G0681* did not form gene clusters (Figure 7A). These four tandem duplications are inferred to have originated from polyploid ancestors, while *GH_D09G0617* and *GH_A09G0681* are believed to have arisen in *G. hirsutum* following its divergence from *G. barbadense*, as they are absent in *G. barbadense* (Figure 7B).

### 2.6. Silence of GhGT61 GhGTs Affected the Disease Resistance of Cotton

**Figure 8:**
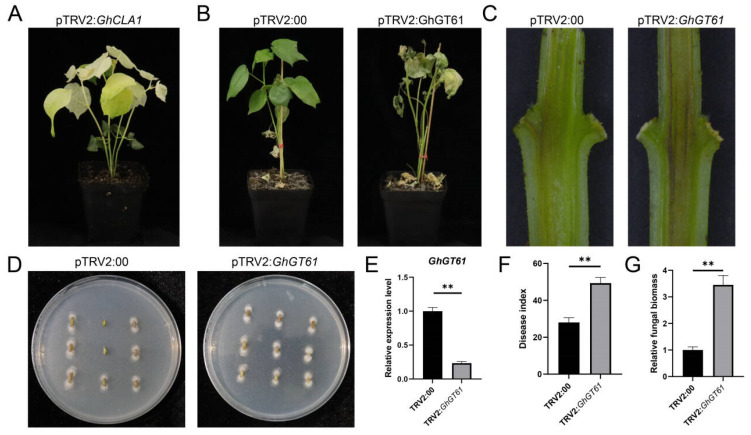
Function of *GhGT61* on the resistance of cotton to *V. dahliae*. (**A**) VIGS-mediated silencing of *GhCLA1* results in an albino phenotype in cotton. (**B**) Phenotype of silenced and normal plants inoculated with *V. dahliae* at 13 dpi. (**C**) Vascular bundle browning observed in longitudinal stem sections of silenced and control plants. (**D**) Fungal biomass recovery experiment in stems of silenced and control plants. (**E**) The expression level of *GhGT61* in silenced and normal plants. (**F**) Disease index of silenced and normal plants at 13 dpi. (**G**). qRT-PCR quantification of fungal biomass in stems in silenced and control plants. The data are presented as mean ± SD (n = 3). Significance is determined using Duncan’s multiple range test, indicated by ** *p* ≤ 0.01.

To elucidate the role of *GhGTs* on the resistance of cotton to Verticillium wilt, Virus-Induced Gene Silencing (VIGS) and disease resistance assays were carried out. The results showed that an albino phenotype was observed at 14 dpi (Figure 8A). Following inoculation with *V. dahliae* 13 days after silencing, plants with suppressed *GhGT61* exhibited markedly reduced resistance to the *V. dahliae* (Figure 8B,F), with a significant reduction in the expression level of *GhGT61* (Figure 8E). A histological analysis of longitudinal sections of cotton cotyledonary nodes revealed pronounced browning in TRV2:*GhGT61* plants compared to TRV2:*00* controls (Figure 8C). The fungal biomass quantification via recovery culture showed significantly higher *V. dahliae* accumulation in stems of TRV2:*GhGT61* plants than in TRV2:*00* controls (Figure 8D,G). These results collectively demonstrate that silencing *GhGT61* enhances cotton susceptibility to *V. dahliae*, underscoring its critical role in mediating resistance to Verticillium wilt.

## 3. Discussion

In recent years, continuous cropping has resulted in a significant increase in Verticillium wilt incidence in the cotton industry, severely impacting cotton yields. The application of modern molecular techniques for identifying resistance-related genes has accelerated the breeding of disease-resistant varieties [4]. Exploring key genes associated with cotton’s resistance to Verticillium wilt and elucidating their mechanism is crucial for developing new cotton germplasm resistant to Verticillium wilt [15]. Recent advancements in sequencing technology and decreasing costs have significantly improved and updated cotton genome sequencing efforts, providing a foundation for studying gene functions at the genomic level [16]. Transcriptome analysis can identify potential genes and molecular mechanisms involved in various biotic stress responses, playing a vital role in elucidating gene function [17]. A more profound comprehension of cotton genomics and genetics opens new pathways for investigating the *GT* family.

Glycosylation is a common modification of plant secondary metabolites and plays various functions, including the regulation of hormone homeostasis, detoxification of exogenous substances, and the biosynthesis and storage of secondary compounds. These processes are facilitated by specific subclasses of the *GT* family [18]. However, there are few studies on the biotic stress of cotton *GTs*. In the present study, we identified that there were eight *GTs* involved in cotton regulation of the defense response to *V. dahliae* in the early infection period. The *GT* family has remained conserved throughout the long-term evolution of cotton, consistent with its evolutionary relationships. Notably, the number of *GTs* in tetraploid cotton is approximately double that of diploid cotton species, further supporting the existence of two diploid ancestors for tetraploid cotton [19].

Gene function is closely linked to the differentiation of the promoter region, which are the combination regions of many transcription factors (TFs) [20]. TFs usually regulate various aspects of plant growth, development, and stress resistance by regulating gene expression in responses to biotic and abiotic stress factors [21]. An analysis of the cis-elements in the promoter regions of these eight *GTs* revealed that the cis-elements with the strongest hormone response were ABA- and MEJA-response cis-elements. This suggests that these *GTs* may be involved in the response of cotton to *V. dahliae* through the ABA or MeJA pathway. Additionally, light-responsive cis-regulating elements account for a large proportion in the eight *GTs*, accounting for 27.336% of the total, indicating that *GTs* may participate in the regulation of light responses in cotton.

At present, the main goal of cotton breeding is to select new high-yield and high-quality varieties resistant to Verticillium wilt [22]. In this study, we analyzed the RNA-Seq data set (PRJNA1121084) of cotton inoculated with *V. dahliae* and found that eight *GTs* were differentially expressed in resistant and susceptible cotton after inoculation with *V. dahliae*, indicating that *GTs* may be involved in the defense process of cotton against *V. dahliae*. Significantly, silencing *GhGT61* compromised plant resistance, establishing its critical role in disease response.

Studies have shown the multifaceted role of *GTs* in plant−pathogen interactions. In *Brachypodium*, a UDP-glycosyltransferase enhances Fusarium resistance through DON detoxification [23], while rice *OsTGAL1* modulates bacterial leaf streak susceptibility via SA glycosyltransferase regulation [24]. *Arabidopsis* UGT73C3/UGT74C4 catalyze immunostimulatory pinoresinol glycosylation [25], in contrast with the immunosuppressive effect of *OsGMT1* overexpression in rice [26]. These functional divergences underscore substantial interspecies variation in *GT*-mediated defense mechanisms. Future investigations should employ transgenic approaches and CRISPR/Cas9-mediated knockout strategies to elucidate functional redundancy and synergistic effects within *GT* families, particularly in Verticillium wilt resistance.

## 4. Materials and Methods

### 4.1. RNA-Seq and qRT-PCR Analysis

The transcriptome data were downloaded from the NCBI database (Project ID: PRJNA1121084) [27]. Data were filtered and quality-controlled using Fastp software, version 0.23.4 [28]. Clean data were aligned to the *G. hirsutum* TM-1 genome (ZJU-improved_v2.1_a1) using HISAT2, and StringTie was used for the read quantification. The gene expression was evaluated using the RPKM method [29]. For qRT-PCR, primers were designed using Primer 5.3 software based on the cDNA sequences of eight candidate genes (Table S1). Expression levels were analyzed using root tissue cDNA as a template. Each sample was replicated seven times, and *GhUBQ7* was used as an internal reference gene. qRT-PCR was performed as described previously [30].

### 4.2. Identification of GTs in Cotton

Genomic data of *G. arboretum* (*Ga*), *G. raimondii* (*Gr*), *G. hirsutum* (*Gh*), and *G. barbadense* (*Gb*) were obtained from the CottonGen database http://www.cottongen.org/ (accessed on 13 August 2024). Homologous sequences were identified using BLAST with an e-value threshold of 1e-5. A total of 21, 21, 41, and 40 *GTs* were retrieved in *Ga*, *Gr*, *Gh,* and *Gb*, respectively [31].

### 4.3. Analysis of Gene Structure and Conserved Motifs

Gene structure analysis was performed using TBtools version 2.149 to identify the exons, introns, and untranslated regions of *GTs* in cotton. Domain analysis was performed using the NCBI conserved domain database (CDD) to determine the domain types and positions. Subsequently, the gene exons, introns, and conserved motifs were visualized using TBtools version 2.149.

### 4.4. Phylogenetic Analysis of GTs in Cotton

Multiple sequence alignments were performed using Clustal W in MEGA X version 11.0.13 with default settings (gap opening penalty = 10, gap separation distance = 4). A phylogenetic tree with 1000 bootstrap replicates was constructed using the neighbor-joining (NJ) method. The tree was visualized and edited using FigTree (http://tree.bio.ed.ac.uk/software/Figtree/ (accessed on 16 August 2024)) [32].

### 4.5. Analysis of Cotton Glycosyltransferase Activity Gene Promoter Region

The 2000 bp upstream DNA sequences of the *GTs* were extracted from the CottonGen database (http://www.cottongen.org/ (accessed on 11 August 2024)). Cis-acting elements were predicted using the PlantCARE (http://bioinformatics.psb.ugent.be/webtools/plantcare/html/ (accessed on 11 August 2024)) [33], and their positions were visualized using TBtools version 2.149.

### 4.6. VIGS of GhGT61

RNA was isolated from cotton roots and reverse-transcribed into cDNA. A 300 bp CDS fragment of *GhGT61* (GH_D12G2902) was amplified using primers with *BamHI* and *EcoRI* restriction sites. The fragment was cloned into the TRV2 vector by homologous recombination and transformed into Agrobacterium GV3101. The transformed TRV2 vector was injected into Z8 with completely flattened cotyledons, and then the transformed seedlings were transferred to a growth chamber at 25 °C with a light/dark photoperiod of 16 h/ 8 h. VIGS efficiency was systematically validated using TRV2: *GhCLA*-positive controls, with successful gene suppression phenotypically confirmed by characteristic leaf albinism at 15 days post-agroinfiltration (DPI). For molecular validation, second true leaves were collected from silenced plants (n = 30 across three biological replicates) for RNA extraction and subsequent RT-qPCR analysis using *GhUBQ7* as the reference gene (2^−ΔΔCt^ method). Following confirmation silencing efficiency, the plants were challenge-inoculated with the defoliating *V. dahliae* strain V592 (1 × 10⁶ spores/mL, root-dip method) [34]. The highly pathogenic strain V592 of *V. dahliae* was provided by Professor Jiafeng Huang from Shihezi University [35], cultured in Czapek Dox liquid medium [36]. Disease progression was monitored at 13 dpi, and the disease index was calculated according to standardized 0–4 grading scales [37]. The fungal biomass was detected by qRT-PCR using fungal-specific ITS1-F primer and *V. dahliae*-specific reverse primer ST-VE1-R, and *GhUBQ7* as the internal reference gene [28]. The fungal recovery test was performed according to the previously described method [38]. After 4 days of culture, the fungal colonies were observed.

### 4.7. Statistical Analysis

All of the experiments were conducted with three independent biological replicates, each containing three technical repetitions. Quantitative data are presented as mean ± standard deviation (SD). Significant differences between wild-type (WT) and treatment groups were determined using Duncan’s multiple range test (SPSS v26.0), with asterisks denoting significance levels: * *p* < 0.05, ** *p* < 0.01. Statistical homogeneity within subgroups was indicated by identical lowercase letters (a, b, c) in the corresponding figures.

## 5. Conclusions

This study identified eight *GTs* that were differentially expressed in cotton varieties susceptible and resistant to Verticillium wilt through RNA-Seq analysis. The analysis of the gene number, chromosome location, and evolution revealed that the *GT* family is conserved in cotton. The presence of ABA- and MeJA-responsive cis-acting elements suggests that these *GTs* may participate in the response of cotton to *V. dahliae* infection through these hormonal pathways. Additionally, the presence of photoresponsive elements in the cis-acting elements of each gene suggests that *GTs* may be involved in the regulation of the cotton photoperiod. VIGS experiments further revealed that *GhGT61* is involved in the resistance process of cotton to Verticillium wilt. This study provides a foundation for further research on the role of *GTs* in cotton and offers insights for breeding efforts aimed at developing more resistant cotton varieties.

## Data Availability

The data used in this study were from the National Center for Biotechnology Information (NCBI), BioProject ID: PRJNA1121084.

## Supplementary Materials

The following supporting information can be downloaded at: https://www.mdpi.com/article/10.3390/ijms26073170/s1.
