# Supplementary material for: The Analysis of the Glycosyltransferase Activity Gene Family in Gossypium hirsutum and Functional Verification of GTs Conferring Resistance to Verticillium Wilt
Source: Int J Mol Sci. 2025 Mar 29;26(7):3170. doi: 10.3390/ijms26073170 (PMC11989804; doi:10.3390/ijms26073170)
Supplement: Supplementary file 1 [file ijms-26-03170-s001.zip › ijms-3522265-supplementary.pdf]

Supplementary Materials

Table S1. List of primers used in this study.

Primer name	Sequence (5'→3')
GH_A04G1083-F	TCTCACACTTGTTCCATCA
GH_A04G1083-R	GTTCACCATTGCTCAATCTTA
GH_A09G0680-F	AAGTGTTGTTGTTGGTCTC
GH_A09G0680-R	ATCATCGTCATCATCATCCT
GH_A09G0681-F	CGAAGAGGAGACGAAGAG
GH_A09G0681-R	AATATAGAGGAAGCACGATGT
GH_A12G2878-F	GCTTCTCCACTGTCTTCA
GH_A12G2878-R	CGAACCGTCACTAACCAT
GH_D01G2351-F	ATTCCTTCTATCATTACCATTCTC
GH_D01G2351-R	ACTCTACCAGCCTCTAATAAG
GH_D09G0616-F	GACGATGATGACGATGATG
GH_D09G0616-R	CTTGCCATTCCAACGATT
GH_D09G0617-F	CGAAGAGGAGACGAAGAG
GH_D09G0617-R	AATATAGAGGAAGCACGATGT
GH_D12G2902-F	GCTTCTCCACTGTCTTCA
GH_D12G2902-R	CGAACCGTCACTAACCAT
GhGT61-F	gtgagtaaggttaccgaattcTTAACTGTAAATAAGAGACTATCAA TCAATGATGGTTTGAAGA
GhGT61-R	cgtgagctcggtaccggatccCTGTCCTTGTTGAGAACTGCTTCCA
ITS1-F	AAAGTTTTAATGGTTCGCTAAGA
ST-VE1-R	CTTGGTCATTTAGAGGAAGTAA
GhUB7-F	GAAGGCATTCCACCTGACCAAC
GhUB7-R	CTTGACCTTCTTCTTCTTGTGCTTG
